# Animal models of diabetes-related shoulder disorders

**DOI:** 10.3389/fvets.2026.1796034

**Published:** 2026-06-26

**Authors:** Weifeng Guo, Long Chen, Licheng Chen, Wanxia Zhang

**Affiliations:** 1 Guangdong Provincial Second Hospital of Traditional Chinese Medicine, Guangzhou, China; 2 Foshan Hospital of Traditional Chinese Medicine, The Eighth Clinical Medical College of Guangzhou University of Chinese Medicine, Foshan, China; 3 The First Dongguan Affiliated Hospital of Guangdong Medical University, Dongguan, China

**Keywords:** animal models, diabetes, frozen shoulder, rotator cuff tendinopathy, shoulder disorders

## Abstract

Diabetic shoulder pathology constitutes a significant complication of diabetes mellitus, characterized by complex pathological mechanisms and a lack of systematic research models. Existing animal models can simulate pathological processes such as frozen shoulder, rotator cuff repair impairment, or natural degeneration at different levels, yet they exhibit certain limitations. These include inconsistency with the metabolic background of human type 2 diabetes, a lack of dynamic observation of multi-tissue collaborative pathologies, and difficulty in replicating the natural clinical disease course. This article analyzes three mainstream animal models: the diabetes-combined frozen shoulder model, the diabetes-combined rotator cuff injury and repair model, and the type 2 diabetes-induced natural degeneration model of the rotator cuff tendon. By comparing the modeling methodologies, pathological features, advantages, and disadvantages of these different models, potential improvements are proposed. An ideal diabetic shoulder model should simultaneously replicate the metabolic characteristics of type 2 diabetes, simulate the progressive and collaborative pathology of multiple shoulder structures (tendon, joint capsule, subchondral bone), and incorporate behavioral assessments for pain and functional impairment. Promoting a paradigm shift in animal models from “single-disease simulation” to “integrated simulation” will provide an important platform for elucidating the pathogenesis of diabetic shoulder and developing novel therapeutic strategies.

## Introduction

Diabetes mellitus poses a significant global public health challenge, with its complications extending beyond traditional cardiovascular and renal diseases to include the musculoskeletal system (1). Clinical studies indicate a substantially increased incidence of shoulder disorders among diabetic patients. Diabetes is recognized as a high-risk factor for rotator cuff injuries, with the prevalence of rotator cuff pathology ranging from 10% to 30% and that of frozen shoulder (adhesive capsulitis) ranging from 20% to 30% (2, 3). These conditions severely impact patients' quality of life and functional recovery, imposing a substantial healthcare burden (4). Shoulder pathology represents a common yet frequently overlooked musculoskeletal complication of diabetes, manifesting as a complex clinical picture that primarily includes rotator cuff tendinopathy, adhesive capsulitis (frozen shoulder), and impaired tendon-to-bone healing (5–7). These disorders not only cause shoulder pain, restricted mobility, and diminished quality of life but also present a significant clinical challenge due to their often insidious progression and suboptimal treatment response within the hyperglycemic milieu (8). Although epidemiological research has firmly established an association between diabetes and an elevated risk of shoulder disorders, the precise pathophysiological mechanisms underlying this link remain incompletely understood (9). It is widely accepted that persistent hyperglycemia disrupts the structural and functional homeostasis of shoulder soft tissues—including tendons, the joint capsule, and the tendon-bone interface—through multiple interconnected pathways. These include the accumulation of advanced glycation end products (AGEs), chronic low-grade inflammation, microangiopathy, and aberrant tissue remodeling (10–12). Research has demonstrated that a hyperglycemic microenvironment impairs tendon-to-bone healing following rotator cuff repair, potentially mediated through mechanisms such as the let-7b-5p/CFTR pathway (13). Currently, existing animal models lack a systematic understanding of the diabetic shoulder as an integrated functional unit undergoing progressive, multi-structural pathology. There is a particular paucity of experimental platforms capable of replicating the natural history of type 2 diabetes mellitus (T2DM) and dynamically observing its synergistic effects on various shoulder structures, including tendons, the joint capsule, and subchondral bone. This gap limits a holistic perspective on the pathogenesis of diabetic shoulder and hinders the development of broad-spectrum therapeutic strategies targeting common pathological mechanisms.

A literature search was conducted in PubMed, Web of Science, and CNKI databases using keywords including “diabetes mellitus,” “shoulder,” “rotator cuff,” “frozen shoulder,” “tendinopathy,” and “animal model.” This search confirmed that only a limited number of *in vivo* studies have concurrently modeled diabetes and shoulder pathology, highlighting a significant gap in the literature.

Consequently, developing animal models that integrate metabolic dysregulation with multi-structural pathological changes in the shoulder is crucial for advancing the investigation into diabetic shoulder disorders. This passage aims to analyze and systematically evaluate established animal models, focusing on their utility in elucidating the integrated pathology of diabetic shoulder and their potential to inform the development of multi-target intervention strategies.

## Diabetes-combined frozen shoulder model (based on joint immobilization)

### Model establishment

The diabetes-combined frozen shoulder model is established using a two-step protocol of “metabolic disturbance + mechanical restriction,” simulating the clinical characteristic of diabetic patients being more prone to joint stiffness and fibrosis. The study by Kim et al. (14) provides a standard paradigm for this model. Adult male Sprague Dawley rats are selected, and diabetes is induced via intraperitoneal injection of streptozotocin (STZ) at a dose of 65 mg/kg. Successful induction of the diabetic model is defined as a sustained blood glucose concentration exceeding 300 mg/dL. Diabetes is successfully induced in rats 3 days after STZ injection. Subsequently, under anesthesia (achieved by intraperitoneal injection of Zoletil 30 mg/kg and Rompun 10 mg/kg), surgical immobilization of the right shoulder joint is performed to induce stiffness: nylon sutures are passed through the scapula and looped around the humeral shaft to achieve complete immobilization of the glenohumeral joint. The immobilization period lasts for 3 weeks, during which the left shoulder joint serves as an untreated internal control. After the immobilization period, the sutures are removed. An intra-articular corticosteroid injection intervention group is added to the diabetic model cohort to further investigate therapeutic effects.

### Pathological features and mechanisms

The core pathological feature of this model is the synergistic worsening effect of diabetes and mechanical immobilization. In the context of diabetes, joint immobilization not only leads to a significant loss of shoulder joint range of motion but, more critically, triggers progressive and irreversible fibrotic thickening of the joint capsule. The joint capsule area in the diabetic model group is increased compared to the non-diabetic group, resulting in reduced joint volume and mechanical restriction. Histologically, Masson's trichrome staining reveals thickening of the joint capsule accompanied by disordered collagen fiber arrangement, significantly increased density, and abnormal extracellular matrix deposition, consistent with the pathological fibrosis driven by AGEs accumulation (14). Molecular mechanism studies indicate that inflammation accompanied by fibrosis is a major pathological feature of frozen shoulder. The binding of AGEs to their receptor (RAGE) activates the nuclear factor kappa B (NF-κB) pathway, exacerbating the local inflammatory response (15). Functional assessments reveal that the external rotation range of motion of the shoulder joint in the diabetic group rats is reduced compared to the non-diabetic immobilized group, and the recovery rate is significantly slower. These findings are consistent with clinical observations that diabetic patients have a higher risk of post-traumatic joint stiffness and a longer recovery period (4). This model validates that corticosteroid intervention can effectively reverse this process. It not only simulates the pathological features of diabetic shoulder arthropathy but also reproduces key aspects of clinical treatment response, providing a solid foundation for clinical translation (16).

### Advantages and limitations of the model

The core advantage lies in its successful simulation of key clinical pathological features: the synergistic effect of hyperglycemia and mechanical restriction exacerbates fibrotic thickening of the shoulder joint capsule and loss of shoulder joint range of motion. This provides a platform for studying the pathogenesis of diabetic shoulder and evaluating the efficacy of interventions such as corticosteroids. However, this model has significant limitations: it is an “acute induction-acute immobilization” accelerated model, which shows a temporal discrepancy from the chronic and insidious progression of the human disease; it focuses primarily on stiffness and generally lacks systematic assessment of pain-related behaviors; and the STZ-induced type 1 diabetes model differs in its metabolic basis from the clinical majority of patients with type 2 diabetes, limiting its applicability.

## Diabetes-combined rotator cuff injury and repair model (based on surgical intervention)

### Model establishment

This model focuses on investigating the impact of diabetes on soft tissue healing capacity. The rotator cuff transection and repair model established by Bedi et al. (12) simulates the repair challenges faced by clinical diabetic patients using a two-step protocol of “metabolic disorder combined with surgical injury.”

First, type 1 diabetes is induced in male Lewis rats in the experimental group via intraperitoneal injection of streptozotocin (STZ, 65 mg/kg), and the hyperglycemic state is confirmed by glucose tolerance testing. A non-diabetic control group was established by injecting citrate buffer only. Subsequently, both diabetic and non-diabetic control animals undergo unilateral supraspinatus tendon transection followed by immediate transosseous suture repair. Postoperative glycated hemoglobin (HbA1c) measurements are used to consistently confirm the metabolic abnormality. Specimens are collected at 1 and 2 weeks postoperatively for systematic evaluation of the biomechanical strength, histomorphology, and molecular pathological changes at the tendon-bone interface. This model successfully reproduces the core pathological features of delayed tendon-bone healing and impaired mechanical performance under diabetic conditions.

### Pathological features and mechanisms

The core pathological feature of this model is a severe reduction in the mechanical strength of the tendon-bone interface and impaired fibrocartilage formation. Histological analysis reveals disordered collagen arrangement, incomplete fibrocartilage band formation, and reduced vascularization at the healing interface in diabetic animals (8). The underlying mechanism involves a vicious cycle driven by hyperglycemia: accumulation of AGEs interferes with collagen assembly and activates the RAGE/NF-κB pathway, leading to chronic inflammation. This inflammatory milieu ultimately impedes the normal formation of a functional tendon-bone interface.

### Advantages and limitations

The primary advantage of this model lies in its accurate simulation of the core clinical problem of impaired tendon-bone healing in the context of diabetes. It provides stable and quantifiable biomechanical and histological evaluation metrics, making it suitable for mechanistic studies and therapeutic screening. Its limitations are as follows: as an acute surgical model, its observation period is short and cannot reflect the chronic progression of the human disease; the STZ-induced type 1 diabetes model differs metabolically from the more common clinical type 2 diabetes; and the model focuses primarily on structural repair, with insufficient assessment of pain and functional behavioral outcomes.

## Metabolic-driven natural degeneration model of diabetic rotator cuff tendon

### Model establishment

The natural progression model of type 2 diabetes-related rotator cuff tendinopathy, developed by Xu et al. (17), systematically simulates the progressive degeneration of the rotator cuff tendon under the long-term influence of clinical type 2 diabetic metabolic disorders. The model employs adult male Sprague-Dawley rats and utilizes a protocol of “high-fat diet-induced insulin resistance combined with low-dose chemical injury” for modeling. Animals are first fed a high-fat diet for 4 weeks to induce insulin resistance and a pre-metabolic syndrome state. Subsequently, a low-dose intraperitoneal injection of streptozotocin (40 mg/kg) is administered to impair pancreatic β-cell function, establishing the core pathophysiology of type 2 diabetes onset. Successful induction of diabetes is defined as three consecutive measurements of fasting blood glucose ≥16.7 mmol/L. After successful modeling, animals are maintained under hyperglycemic conditions without any surgical intervention to the shoulder joint. Serum and shoulder joint specimens are collected in batches at weeks 2, 4, 8, and 12. A comprehensive assessment is performed using enzyme-linked immunosorbent assay, multimodal histological staining, biomechanical tensile testing, and real-time quantitative PCR to evaluate the systemic impact of varying durations of diabetes on the structure, composition, mechanical properties, and molecular microenvironment of the supraspinatus tendon. This model is the first to systematically construct, in a temporal dimension, the progressive pathology of diabetes-induced rotator cuff tendon degeneration, from early collagen disorganization to late-stage structural disruption and mechanical failure.

### Core pathological mechanisms

The core pathological feature revealed by this model is the progressive degeneration of the rotator cuff tendon induced by diabetes, characterized by time-dependent exacerbation of collagen structure disruption, abnormal tissue remodeling, and loss of biomechanical integrity. From sparse collagen arrangement and micro-tears appearing 2 weeks post-modeling, it progresses to evident collagen rupture, scar hyperplasia, and neovascularization by week 12. Concurrently, the ultimate load of the tendon decreases significantly starting from week 8, showing a significant negative correlation with serum levels of AGEs. The intrinsic mechanism involves chronic abnormal remodeling driven by the hyperglycemic microenvironment: persistent hyperglycemia leads to AGEs deposition, which directly compromises tendon mechanical properties by disrupting collagen cross-linking and activates inflammatory pathways. Simultaneously, it triggers a cascade of dysregulated repair responses, including upregulated expression of pro-fibrotic proteins (e.g., Biglycan), increased activity of matrix-degrading enzymes (e.g., MMP-3), release of pro-angiogenic factors, and aberrant expression of adipogenic genes (e.g., FABP4). These processes collectively establish a chronic, low-grade pathological repair microenvironment. This disrupts the homeostasis of the tendon extracellular matrix and prevents the maintenance of normal mechanical structure, thereby simulating the progressive degenerative process of human diabetic rotator cuff tendinopathy.

### Advantages and limitations

The prominent advantage of this model lies in its excellent clinical relevance: it accurately simulates the diabetic metabolic environment and provides a relatively complete representation of the progressive natural history of diabetic rotator cuff tendon degeneration, from early microscopic degradation to late-stage functional loss, offering an ideal window to elucidate the long-term patterns of disease onset and development. However, its significant limitations are primarily reflected in the lengthy research cycle, high cost, and operational complexity, which impose considerable demands on experimental resources. Furthermore, as a non-injury observational model, it fails to incorporate key clinical events such as rotator cuff tears, precluding the study of the interaction between injury and metabolic abnormalities. Moreover, in this model, the T2DM rats exhibited significantly lower body weight than the control group, which may have reduced mechanical loading on the tendon and partially underestimated the true effect of hyperglycemia. Additionally, the inherent differences in shoulder joint mechanical loading between animals and humans remain a persistent translational bottleneck.

## Discussion

The establishment and development of animal models for diabetic shoulder pathology have evolved from simulating single pathological phenotypes to exploring complex metabolic-structural interactions. The three core models reviewed in this article—the “immobilization model” focusing on joint stiffness, the “surgical model” focusing on repair impairment, and the “metabolic model” simulating natural degeneration—reveal the specific damage inflicted by the hyperglycemic environment on shoulder joint tissues from different dimensions. Collectively, these models validate the pathological characteristics of diabetic shoulder, demonstrating that persistent hyperglycemia disrupts shoulder tissue homeostasis through multiple mechanisms, including the AGEs-RAGE axis, chronic inflammation, and abnormal repair.

However, the current model system exhibits certain limitations. Firstly, the simulated pathological features are relatively singular. The “shoulder immobilization model” struggles to reflect rotator cuff degeneration, while the “metabolic model” cannot simulate acute injury to shoulder tissues. This discrepancy does not align with the clinical reality where patients often present with multi-structural pathologies simultaneously. Secondly, most models utilize STZ-induced hyperglycemia, which differs fundamentally from type 2 diabetic patients in aspects such as insulin resistance and lipid metabolism disorders. Finally, existing research predominantly emphasizes structural degeneration, including tissue morphology and biomechanics, while neglecting functional degeneration such as pain and functional impairment. This oversight weakens the clinical translational value of these models.

Consequently, future animal models for diabetic shoulder should aim for integration as their objective. New models should employ methods such as high-fat diets and genetic modification to simulate the complete metabolic profile of type 2 diabetes. Building on this foundation, interventions like surgery and joint fixation should be combined to synchronously induce multiple pathological changes, including joint capsule fibrosis, tendon degeneration, and impaired tendon-bone healing. Regarding model evaluation, while continuing to prioritize structural degeneration, such as tissue morphology and biomechanics, equal emphasis must be placed on functional degeneration, including pain and functional impairment, to construct a comprehensive assessment system.

## Conclusion

Current animal models for diabetic shoulder can simulate the pathological process to a certain extent but are limited by the singularity of the simulated pathological process and the disconnection from the simulated metabolic background. Future efforts require the development of “integrated models” capable of synchronously simulating the metabolic characteristics of type 2 diabetes, progressive multi-structural pathologies, and pain-related functional impairment.

## Data Availability

The original contributions presented in the study are included in the article/supplementary material, further inquiries can be directed to the corresponding authors.
